# Improvement in tuberculosis infection control practice via technical support in two regions of Ethiopia

**DOI:** 10.1186/s12879-018-3459-0

**Published:** 2018-11-12

**Authors:** Asfaw Ayalew, Zewdu Gashu, Tadesse Anteneh, Nebiyu Hiruy, Dereje Habte, Degu Jerene, Genetu Alem, Ilili Jemal, Muluken Melese, Pedro G. Suarez

**Affiliations:** 1 Management Sciences for Health, Help Ethiopia Address the Low Performance of Tuberculosis (HEAL TB) Project, Bole Sub City, Kebele 02, House Number 708, PO Box 1157, Code 1250 Addis Ababa, Ethiopia; 2 Amhara Regional Health Bureau, PO Box 495, Bahir Dar, Ethiopia; 3 Oromia Regional Health Bureau, PO Box 24341, Addis Ababa, Ethiopia; 4 Management Sciences for Health, Health Programs Group, 4301 North Fairfax Drive, Suite 400, Arlington, VA 22203 USA

**Keywords:** TB infection control, TB transmission, TB prevention, Health care workers, Ethiopia

## Abstract

**Background:**

Globally recommended measures for comprehensive tuberculosis (TB) infection control (IC) are inadequately practiced in most health care facilities in Ethiopia. The aim of this study was to assess the extent of implementation of TB IC measures before and after introducing a comprehensive technical support package in two regions of Ethiopia.

**Methods:**

We used a quasi-experimental design, whereby a baseline assessment of TB IC practices in 719 health care facilities was conducted between August and October 2013. Based on the assessment findings, we supported implementation of a comprehensive package of interventions. Monitoring was done on a quarterly basis, and one-year follow-up data were collected on September 30, 2014. We used the Student’s *t*-test and chi-squared tests, respectively, to examine differences before and after the interventions and to test for inter-regional and inter-facility associations.

**Results:**

At baseline, most of the health facilities (69%) were reported to have separate TB clinics. In 55.2% of the facilities, it was also reported that window opening was practiced. Nevertheless, triaging was practiced in only 19.3% of the facilities. Availability of an IC committee and IC plan was observed in 29.11 and 4.65% of facilities, respectively. Health care workers were nearly three times as likely to develop active TB as the general population. After 12 months of implementation, availability of a separate TB room, TB IC committee, triage, and TB IC plan had increased, respectively, by 18, 32, 44, and 51% (*p* < 0.001).

**Conclusions:**

After 1 year of intervention, the TB IC practices of the health facilities have significantly improved. However, availability of separate TB rooms and existence of TB IC committees remain suboptimal. The burden of TB among health care workers is higher than in the general population. TB IC measures must be strengthened to reduce TB transmission among health workers.

## Background

Tuberculosis (TB) is one of the major infectious diseases that have tested the knowledge and wisdom of mankind. Innovations related to TB diagnosis, prevention, and treatment have lagged behind other technological advancements. After isolation of the tubercle bacillus, it took 38 years to invent a preventive vaccine and 61 years to discover the first anti-TB drug [1, 2]. The death toll from TB is enormous, and it continues to claim millions of lives throughout the world, even though simple preventive measures exist. Through airborne transmission, its favored mode of dissemination, TB has managed to sustain itself for millennia [3, 4].

Against all odds, remarkable progress has been made in recent times through formulation and implementation of effective strategies like the Directly Observed Treatment, Short course (DOTS) and Stop TB strategies, which greatly contributed to meeting the TB-related target of the Millennium Development Goals (MDGs) of halting and beginning to reverse the TB epidemic [5]. On one hand, between 2000 and 2015, TB mortality declined by 34% and the number of deaths averted as a result of TB treatment was estimated at 49 million. On the other hand, TB remained one of the top 10 killer diseases in 2015. Moreover, increasing TB mortality was seen in Congo and the Democratic People’s Republic of Korea [6].

Ethiopia is one of the 30 high-TB-burden countries that have shown a substantial reduction in TB incidence and mortality since 2000. The estimated decline in the incidence rate as of 2010 was 6.7%. In 2015, the estimated TB incidence was 191,000, while the actual notified TB cases were 137,960. In the same year, 599 cases of drug-resistant TB (DR-TB) were notified. Of the notified TB cases, 8% were people living with the human immunodeficiency virus (HIV) [6].

In low-income countries such as Ethiopia, patients and visitors tend to congregate in health facilities’ corridors and waiting areas. This congestion is due to uncontrolled population growth and an increasing number of health care seekers, on one hand, and a shortage of health care workers (HCWs) and limited number of health care facilities (HCFs), on the other hand [7]. This overcrowding, in turn, supports airborne nosocomial transmission of TB to HCWs, patients, and even visitors [8]. Highly vulnerable people, such as children, undernourished people, and immunocompromised people, are among the health service seekers in HCFs, which is an opportunity for TB to continue its spread. The emergence and rapid spread of drug-resistant mycobacterial strains, as well as its interaction with HIV, have heightened the demand for effective infection control (IC) interventions at all levels [9–11].

IC, in the TB context, aims at reducing the transmission of TB within populations and relies on a set of managerial, administrative, environmental, and personal protective interventions. When such measures are not implemented, the risk of TB (both drug susceptible and drug resistant) dramatically increases, in some circumstances to epidemic proportions. Inpatient outbreaks of DR-TB among people living with HIV have been reported in Africa and Europe [12, 13]. Implementing IC measures, however, is known to reverse the airborne threat [9]. The TB IC hierarchy of measures is so crucial that the World Health Organization (WHO) has issued a standalone policy document and implementation guidelines for member countries’ action. TB IC has also been recommended in important global documents [8, 14–16].

Ethiopia developed its first national TB IC guidelines based on international recommendations in 2009—in the same year that the relevant WHO documents were published [5, 9]. The Federal Ministry of Health has trained a number of health care workers using a TB IC curriculum. Still others were trained in comprehensive TB/HIV services as well as programmatic management of DR-TB, both of which have incorporated TB IC topics [17, 18].

The Help Ethiopia Address the Low Performance of TB (HEAL TB) project led by Management Sciences for Health (MSH) implemented a comprehensive TB control program, one of the components being TB IC. Capacity building for health managers and health workers, supportive supervision, and technical and material support were among the interventions geared toward improving TB IC in HCFs. It is evident that there is little research, particularly intervention studies, about TB IC in low-income settings. The objective of this study was to compare the implementation of TB IC measures before and after introduction of a comprehensive technical support package in two regions of Ethiopia.

## Methods

### Setting

Ethiopia is the second most populous nation in Africa, with a population close to 99 million. It is administratively composed of nine regional states and two city administrations. Under each regional state there are a number of zonal administrations (also called zones), and under each zone are several *woredas* (districts) [19].

In 2017, there were 22,807 TB notified cases out of the 21.1 million population in Amhara, and 43,321 notified TB cases out of 35.8 million population in Oromia Region [20].

The study employed a pre- and post-intervention design built upon project implementation. All of the project HCFs were included in the study.

Funded by the United States Agency for International Development (USAID), the HEAL TB project supported the government of Ethiopia in addressing many of the major challenges posed by TB. The project began in July 2011 in two of the most populous regional states in the country: Oromia and Amhara, with a total population of 57 million. Before the intervention, HEAL TB conducted a baseline assessment in all HCFs of the selected 11 zones. Twenty-two hospitals and 697 health centers were included in the assessment, which ran from August through October 2013. Basic information on TB IC measures was collected.

### Intervention

The intervention package consisted of capacity building, provision of standard operating procedures (SOPs), regular supportive supervision, and program review meetings. The project initially trained woreda TB focal persons on the basics of TB IC and on supervisory skills so that they could provide technical support to HCWs. The woreda TB focal person supervises, on average, five HCFs under his/her catchment once every three months, using supervision checklists and TB SOC indicators. Both templates address TB IC issues. Based on the findings of the supervision, the woreda TB focal person gives on-site feedback and subsequently follows the implementation status of the action plan developed during the last visit. In addition to the woreda TB focal persons, HCWs were also trained using a TB IC curriculum. Different SOPs appropriate to the service delivery points were developed and posted on the wall, starting with the triage room continuing all the way to the laboratory. TB program review meetings were held biannually; during the meetings, woreda TB focal points presented the performance of the HCFs in their respective woredas over the previous six months. In such meetings, TB IC is a major topic that is thoroughly discussed, and improvement plans are formulated.

### Data collection instruments

Two types of checklists were used, one for baseline assessment and another for quarterly monitoring. The baseline checklist is a comprehensive tool of eight pages with 171 items structured into different thematic areas, including TB IC. The TB IC questionnaire asks about the (1) availability of a separate TB room, (2) opening of windows during consultation hours, (3) presence of cough triage, (4) TB IC committee, (5) TB IC plan, and (6) number of HCWs who developed active TB in the previous year. The quarterly monitoring instrument also contains a subsection on TB IC specifically referring to the presence of a functional multidisciplinary team/infection prevention committee, revised IC plan, triage/cougher prioritization, and separate TB room. Both tools were developed based on consultation with stakeholders and adaptation of international TB standards [21, 22]. Taking into account the large number of HCFs under project support, it was decided that the number of TB IC indicators should be kept to the minimum, while ensuring robustness, for the sake of efficient resource utilization.

### Data collectors and data collection procedures

For the baseline assessment, the data collectors were HEAL TB employees who were medical doctors, health officers, and specialist laboratory professionals, all with public health experience. Training on the baseline assessment tools was provided for two days, before their departure to the field. Observation, review of documents, and interviews were the approaches used to collect the baseline data. Information about the number of HCWs who developed active TB was obtained from the TB registers kept in TB rooms. For this purpose, HCWs were defined as all people working in HCFs.

For quarterly monitoring, the trained woreda TB focal persons were in charge of data collection from their respective catchment HCFs. During the visits, they used the SOC tool, which required them to interview HCF TB focal persons about the status of TB IC activities, to review documents (minutes of infection prevention/IC committees), and to observe practices (prioritization of people with cough; status of the TB room).They spent on average half a day in each HCF to collect relevant TB program data. The following four TB IC indicators were monitored every three months: existence of (1) TB IC committee, (2) triage, (3) separate TB room, and (4) revised/updated TB IC plan. Data were collected from all 719 health facilities at baseline and every quarter thereafter.

## Data management and analysis

A data entry template in the Census and Survey Processing System (CSPro), version 4.1 (US Census Bureau, ORC Macro International, and Serpro SA), was prepared, and data were entered into the template immediately after data collection. We checked the data for consistency and completeness by running frequency tables and cross-tabulations.

The data entered into CSPro 4.1 were exported to Stata 11 (College Station, TX: StataCorp., LP), where data cleaning, editing, and consistency checking were performed. Frequencies and percentages were computed to describe the data and compare TB IC practices before and after the program was implemented. We used the Student’s *t*-test to determine significant differences between baseline and post-intervention data. The chi-squared test (Table 1) was also utilized to test for associations between the two regions as well as between hospitals and health centers (Tables 2 and 3).

**Table 1:** Comparison of the TB IC performance before and after intervention

Variables	Before intervention % (*N* = 719)	After intervention % (*N* = 719)	*P*-value (Before after t-test)
Separate TB room	69.1	87.1	*P* < 0.001
Cough triage	19.3	63.0	*P* < 0.001
IP/TB IC committee	29.1	61.1	*P* < 0.001
TB IC Plan	4.7	56.1	*P* < 0.001

**Table 2:** TB IC performance at baseline and after intervention by region

Characteristics	Baseline			After Intervention		
	Region		*P*-value (Chi-square)	Region		*P*-value (Chi-square)
	Amhara (*n* = 306)	Oromia (*n* = 413)		Amhara (*n* = 306)	Oromia (*n* = 413)	
% Cough triage	17.4	21.8	0.31	84.2	46.7	< 0.01
% IP/TB IC committee	57	14.7	< 0.01	71.2	53.6	< 0.01
% TB IC Plan	2.9	6.3	0.16	67.3	48.0	< 0.01

**Table 3:** TB IC performance at baseline and after intervention by type of health facility

Characteristics	Baseline			After Intervention		
	Type of Health Facility		*P*-value (Chi-square)	Type of Health Facility		*P*-value (Chi-square)
	Health Center (*n* = 697)	Hospital (*n* = 22)		Health Center (*n* = 697)	Hospital (*n* = 22)	
% Cough triage	18.8	47.8	< 0.01	61.5	91.7	< 0.01
% IP/TB IC committee	28.6	56.5	< 0.01	60.4	79.2	0.10
% TB IC Plan	3.6	34.8	< 0.01	55.2	83.3	0.01

## Ethical considerations

We received ethical approval from the ethics committees of Amhara and Oromia Regional Health Bureaus to analyze the routine data and disseminate the findings. We used health facility level reports for this analysis with the consent of the reporting institutions. No patient identifiers were included in the routine reports.

## Results

At baseline, separate TB clinics existed in the majority of health facilities (69.1%). HCWs assigned to outpatient departments were reported to be working with open windows in 55.2% of the HCFs. However, coughing patients were prioritized for TB services in only 19.41% of the health facilities. Infection prevention committees and TB IC plans existed in only 29.11 and 4.65% of the assessed HCFs, respectively. There were no statistically significant differences between Amhara and Oromia regions regarding the practice of cough triage (*p* = 0.31) and existence of a TB IC plan (*p* = 0.16).However, regional differences in existence of TB IC committees (*p* < 0.01) did attain statistical significance (Table 2). Furthermore, hospitals differed from health centers significantly in terms of cough triage, TB IC committees, and TB IC plan availability (p < 0.01) (Table 3).

Sixty-one of the 8667 HCWs had developed active TB in the year preceding this assessment, making the annual incidence rate 704/100,000. Using WHO’s 2012 TB incidence estimation for Ethiopia, which was 247/100,000, we calculated the incidence rate ratio to be 2.85 (for all forms of drug-susceptible TB, not only new cases). The incidence risk ratio was not age standardized; the rates were compared to the general population based on the WHO estimate, which is not age standardized.

Health care workers with TB were identified according to the national diagnostic algorithms, which consisted of sputum smear microscopy (the algorithm was recently revised, so that HCWs are tested with GeneXpert assay), radiography, and cytology—as indicated.

After 12 months of implementation, the number of HCFs with TB IC committees increased to 61%, and prioritized service for coughers had been put in place in 63% of the HCFs (*p* < 0.001). Furthermore, 56% of HFs had written TB IC plans, and 87% had designated separate TB rooms, which was significantly higher than the situation before intervention (p < 0.001) (Table 1).

Post-intervention, statistically significant differences occurred between Amhara and Oromia regions pertaining to availability of all the TB IC measures (*p* < 0.01). Moreover, with the exception of existence of TB IC committees (*p* = 0.10), hospitals differed from health centers significantly in terms of cough triage (p < 0.01) and availability of TB IC plans (*p* = 0.01).

## Discussion

Before the technical support and comprehensive package of interventions, the majority of the health facilities lacked basic work procedures and policies to facilitate smooth implementation of TB IC. After intervention, availability of TB IC plans and use of triage showed marked improvement, while existence of TB IC committees and separate TB rooms improved modestly.

When comparing HCFs in Amhara with those of Oromia Region, the former did well post-intervention in availability of TB IC plans and triage, whereas the latter performed better in existence of TB IC committees. In addition, hospitals performed better than health centers in all the TB IC indicators, which can be explained by a relatively good start at baseline, presence of trained HCWs in sufficient number, and extra supervisory support from different stakeholders due to hospitals’ physical accessibility.

According to the WHO directive, facility-level TB IC implementation begins with assignment of a coordinating body. Health facility risk assessment, formulation of a TB IC plan, and implementation of the plan should follow in that order [9]. In our baseline assessment, however, this logical order was not evident. Only 29% of the facilities had functional IP committees, meaning that the great majority of the facilities lacked a responsible body to combat TB transmission. Without this committee, it is not possible to do facility assessments and to plan TB IC activities, in line with the guidelines. The literature is scanty regarding availability of infection prevention/TB IC committees. Ogbonnaya et al. reported that 16.7% of the assessed TB/HIV implementing health facilities in Nigeria had IC committees [23]. This figure is lower than what we found at baseline in Ethiopia. A 32% improvement was seen post-intervention.

The least-implemented TB IC measure was the facility-level IC plan, which was found in only 4.7% of the HCFs. This also brings to light that 24.3% of the HCFs reported as having IC committees failed to develop plans, bringing into question their functionality. In sub-Saharan Africa, availability of TB IC plans in health facilities ranges from none to 77% [24, 25]. In Mozambique, it was 48% [26] and in Uganda, 31% [27]. Our baseline finding lies in between and was increased to 56% after intervention. Unavailability of a TB IC plan implies poor managerial activity and attention. It is very difficult to implement what has not been planned.

Close to 80% of the assessed facilities did not provide prioritized services for presumptive TB patients. Functional triage availability is reported from Nigeria [23] and South Africa [28] as 16.7, and 26%, respectively. In a 2012 study involving nine countries in sub-Saharan Africa, it ranged from 5 to 93% [25]. At baseline, triage availability in Ethiopia was a bit higher than the reported figure from Nigeria but modestly lower than in South Africa and within the range of figures in sub-Saharan Africa. In such a situation, generation of infectious droplet nuclei and contamination of the environment are facilitated; HCWs, visitors, and other patients become highly exposed.

Screening of all health facility care-seekers for TB symptoms, with subsequent isolation and/or fast tracking for services are triage functions and an essential component of administrative control. It is generally agreed that triage is the first defense mechanism against TB transmission in HCFs. Rapid diagnosis and effective TB treatment are the hallmarks of TB IC. Establishment of triaging had increased to 63% of the HCFs by the end of the intervention year.

At baseline, HCWs in half of the assessed facilities opened window(s) during consultation hours and thereafter. This practice was indicative of individuals’ precautions rather than a concerted IC effort, given the low level of availability of TB IC committees and plans. Moreover, awareness among HCWs has been raised by their attending trainings related to TB, HIV, and DR-TB. Reports of increased awareness of HCWs (clinicians) about IC were recently demonstrated in Addis Ababa and in northwest Ethiopia in a survey of knowledge, attitudes, and practices [29, 30].

In nearly 70% of the health facilities, TB rooms were standalone. This should not be counted as a strength, however, since all service delivery points are required to have separate rooms as a matter of health service standards. It also implies that the remaining one-third of HCFs are providing TB service together with other services, compromising space and ventilation. In South Africa, dedicated TB rooms were available in only 31% of HCFs [25]. Through project support in Ethiopia, it was possible to increase the level from the baseline of 69 to 87%.

Poor implementation of IC measures is reflected in HCWs’ acquisition of TB disease, as this study showed. Sixty-one HCWs acquired TB in 1 year. They were about 2.85 times more likely to acquire active TB disease than the general population. Reports of TB among HCWs from sub-Saharan African countries [24] and resource-rich countries [31] show disparities. The TB case notification rate in HCWs of Malawi was 3.2% as compared to 1.8% for primary schoolteachers [32]. In South Africa, Claassens et al. stated that the standardized incidence ratio of smear-positive pulmonary TB in primary health care workers was more than double that of the general population [28]. A study in 2013 in the same country showed a 10% DR-TB rate among physicians with pulmonary TB diagnosis, which the researchers attributed to lack of effective IC work practices combined with negative attitudes of administrators [33]. A recent meta-analysis by Baussano et al. indicated that the stratified pooled estimates for annual TB incidence risk ratio for countries with high TB incidence (> 100 cases/100,000) is 3.68, whereas it is 2.42 for low-incidence (< 50 cases/100,000) and intermediate-incidence (50–99 cases/100,000) countries. The overall estimate of annual TB incidence risk ratio was 2.97, which is comparable to our finding of 2.85. They concluded that HCWs’ risk of acquiring TB was higher than that of the general population across the globe and that effective TB IC measures could decrease annual TB incidence among HCWs by as much 81, 27, and 49% in countries with high, intermediate, and low TB incidence, respectively [34]. Most, if not all, countries have long recognized TB as an occupational disease [9, 35].

It is worth noting the following limitations of the study. We were not able to present data regarding the cross-ventilation status of outpatient departments and active TB disease among HCWs to make comparison with the baseline findings, since our quarterly data source did not capture these items. Furthermore, lack of randomization in quasi-experimental design makes it difficult to control for confounding variables, and hence statistical association may not necessarily imply causal association. Similarly, we used Student’s *t*- and chi-squared tests, which do not allow adjustment for confounders. Moreover, we relied on facility-level registers to collect data about the number of HCWs with TB, which might miss those being treated outside the HCF; hence there is a possibility of underreporting.

## Conclusions

Building the capacity of district TB focal persons to provide supportive supervision to health facilities, together with the other comprehensive TB program support, was instrumental in improving the TB IC situation of health facilities. Huge gaps in implementation of the recommended TB IC practices at health facility level can be narrowed, if systematized basic capacity building can be provided. The burden of TB among HCWs is higher than the prevalence in the general population. There is a need to further strengthen infection prevention/TB IC committees in order to plan and implement the hierarchy of TB IC activities and reduce TB transmission among patients and HCWs.

## Abbreviations

DR-TB: Drug-resistant tuberculosis
HCF: Health care facility
HCW: Health care worker
HIV: Human immunodeficiency virus
IP: Infection prevention
SOC: Standard of care
TB IC: Tuberculosis infection control
TB: Tuberculosis
USAID: United States Agency for International Development
WHO: World Health Organization
